# Head and neck radiotherapy on the MR linac: a multicenter planning challenge amongst MRIdian platform users

**DOI:** 10.1007/s00066-021-01771-8

**Published:** 2021-04-23

**Authors:** Madalyne Chamberlain, Jerome Krayenbuehl, Janita E. van Timmeren, Lotte Wilke, Nicolaus Andratschke, Helena Garcia Schüler, Stephanie Tanadini-Lang, Matthias Guckenberger, Panagiotis Balermpas

**Affiliations:** Department of Radiation Oncology, University Hospital Zurich, Zurich, Switzerland

**Keywords:** MR-adaptive treatment planning, Step-and-shoot IMRT, MR-guided radiation therapy, Treatment plan comparison, Low field magnetic resonance imaging

## Abstract

**Purpose:**

Purpose of this study is to evaluate plan quality on the MRIdian (Viewray Inc., Oakwood Village, OH, USA) system for head and neck cancer (HNC) through comparison of planning approaches of several centers.

**Methods:**

A total of 14 planners using the MRIdian planning system participated in this treatment challenge, centrally organized by ViewRay, for one contoured case of oropharyngeal carcinoma with standard constraints for organs at risk (OAR). Homogeneity, conformity, sparing of OARs, and other parameters were evaluated according to The International Commission on Radiation Units and Measurements (ICRU) recommendations anonymously, and then compared between centers. Differences amongst centers were assessed by means of Wilcoxon test. Each plan had to fulfil hard constraints based on dose–volume histogram (DVH) parameters and delivery time. A plan quality metric (PQM) was evaluated. The PQM was defined as the sum of 16 submetrics characterizing different DVH goals.

**Results:**

For most dose parameters the median score of all centers was higher than the threshold that results in an ideal score. Six participants achieved the maximum number of points for the OAR dose parameters, and none had an unacceptable performance on any of the metrics. Each planner was able to achieve all the requirements except for one which exceeded delivery time. The number of segments correlated to improved PQM and inversely correlated to brainstem D_0.1cc_ and to Planning Target Volume1 (PTV) D_0.1cc_. Total planning experience inversely correlated to spinal canal dose.

**Conclusion:**

Magnetic Resonance Image (MRI) linac-based planning for HNC is already feasible with good quality. Generally, an increased number of segments and increasing planning experience are able to provide better results regarding planning quality without significantly prolonging overall treatment time.

**Supplementary Information:**

The online version of this article (10.1007/s00066-021-01771-8) contains supplementary material, which is available to authorized users.

## Introduction

In recent years various advances in image-guided radiotherapy (IGRT) have led to improved precision and accuracy in radiation treatment delivery for head and neck cancer (HNC) [1–3]. Three-dimensional image guidance for patient setup allows corrections and could also reduce failures caused by anatomical changes of tumor or other organs, e.g., in case of weight changes during the 6–7-week treatment [4, 5]. Furthermore, the ultimate goal of all novel radiotherapy application techniques is reduction of toxicity, which has already been demonstrated for step-and-shoot intensity-modulated radiotherapy (IMRT) in the past [6]. The vast majority of linear accelerators (linacs) used for HNC treatment nowadays have integrated cone-beam computed tomography (CBCT) or, in the case of tomotherapy, megavoltage (MV) computed tomography (CT) for image guidance [1, 7]. Although these CT scans can detect some anatomical changes and allow patient positioning based on radiopaque structures like bone or metal fiducials, image guidance is generally suboptimal due to low soft tissue contrast, artefacts (e.g., caused by denture or internal fixation plates), and increased noise level. Furthermore, x‑ray-based IGRT leads to additional dose exposure in this sensible region, leading to enhanced deterministic and stochastic risks like cataracts and secondary malignancies, especially if daily imaging is needed [8]. Hybrid platforms consisting of a linac with integrated magnetic resonance imaging (MRI) have gained approval for clinical use only in the last few years, allowing superior soft tissue contrast and live imaging during beam application, without additional exposure to ionizing radiation [9]. These features could lead to the future establishment of MR linacs as the optimal platforms to treat diseases like HNC. In such cases, a relatively high radiation dose has to be applied with a steep gradient to low x‑ray contrast organs at risk (OARs) like salivary glands and pharyngeal constrictor muscles. For these patients both coverage of the planning target volume (PTV) and maximal sparing of OARs are crucial, as they are usually treated with full curative intent and late sequelae with impairment of life quality are a common problem after head and neck irradiation.

In this study, we evaluated the different planning approaches used amongst MRIdian (MRIdian System; ViewRay Inc. Oakwood Village, OH) users. We aimed to investigate application features and feasibility of this approach, with the ultimate goal to improve our plan quality and consequently implement the benefits of MR linac technology in standard routine for the majority of HNC cases in the near future.

## Methods

### Case selection

**Fig. 1:**
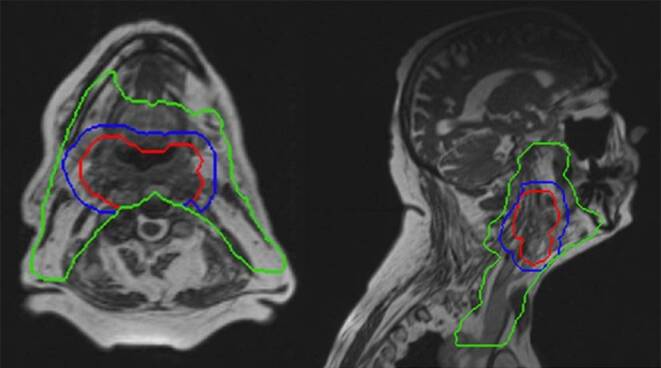
Planning MRI image and PTV contours of the clinical case used in the planning challenge. Three dose levels depicted in *red* (70 Gy), *blue* (59.4 Gy), and *green* (54 Gy). *MRI* magnetic resonance imaging, *PTV* planning target volume

A pre-contoured real-life case of a patient with cT4b cN1 p16-negative squamous cell carcinoma of the posterior oropharyngeal wall was distributed to all participating centers through an online platform. Contouring of high- and intermediate-risk target volumes and neck lymphatics was performed according to international recommendations [10, 11]. An approach according to the guidelines published by Gregoire et al. [11] for clinical target volume (CTV) definition and Biau et al. [10] for the selection of the lymph node target volumes to be irradiated was used. Briefly, a Gross Target Volume (GTV)-to-CTV margin of 10 mm cropped for anatomic boundaries (e.g., air, bone) was adopted for CTV2 (59.4 Gy) and of 5 mm for CTV1 (69.96 Gy). The CTV-to-PTV margins amounted to 3 mm for all three PTVs [12]. The consensus guidelines published by Brouwer et al. [13] were used for definition of OARs. Representative images can be seen in Fig. 1. The prescription was on three dose levels with a simultaneous integrated boost (SIB) technique in 33 factions: 69.96 Gy should be applied to the high-risk volume (PTV1), 59.4 Gy to the intermediate-risk volume (PTV2), and 54 Gy to the “elective,” low-risk volume (PTV3). The patient signed informed consent for data and image use (including the MRI images presented here) according to institutional standards.

The planning MRI consisted of an MRI simulation image in the treatment position performed on the MRIdian system (0.35T). One imaging sequence is currently available on the MRIdian system, which is a True FISP, which gives a mixture of T2-/T1-weighted contrast. The planning MRI was a high-resolution (1.5 mm × 1.5 mm × 1.5 mm resolution) MRI image with a field size of 50 cm × 45 cm × 43 cm, which gives an acquisition time of 172 s. A CT simulation image was also included to use for dose calculation, which was imaged with 120 kV and 3‑mm slices in the treatment position. The patient was positioned on a head coil which was placed over a head rest, then a thermoplastic mask was placed over the patient and these items, and finally, the top head coil was positioned. An example of the positioning can be viewed in Supplementary Fig. 1.

### Platform and planning specifications

All of the important planning constraints were distributed to the participating centers before planning and can be found in Supplementary table 1.

This treatment planning challenge was organized by ViewRay and held on the ProKnow (ProKnow LLC., Sanford, FL, USA; https://proknowsystems.com/) online platform. Participants had 1 month to download the dataset from the ProKnow platform and create a treatment plan for this case. All plans were created using the ViewRay treatment planning system (TPS) which uses a Monte Carlo (MC) dose calculation algorithm taking into account the magnetic field of 0.35 T. Inverse optimization using objectives and constraints was used to create step-and-shoot IMRT plans [14]. The CT was registered to the planning MRI in the TPS using deformable image registration with default settings. The electron density of this deformed CT image was then used for dose calculation of all submitted plans. Once a plan had been created, participants were able to upload this plan onto the ProKnow platform. Afterwards, the plans were analyzed based on a scoring scheme created in conjunction with ViewRay. Each plan was then awarded a score out of 150 points. The delivery time (gantry and multileaf collimator [MLC] and beam-on time) of the plan had to be 20 min or less according to the treatment planning system, and the plans were to be calculated with the beam model of the local department of the planner.

### Challenge criteria and parameters

**Fig. 2:**
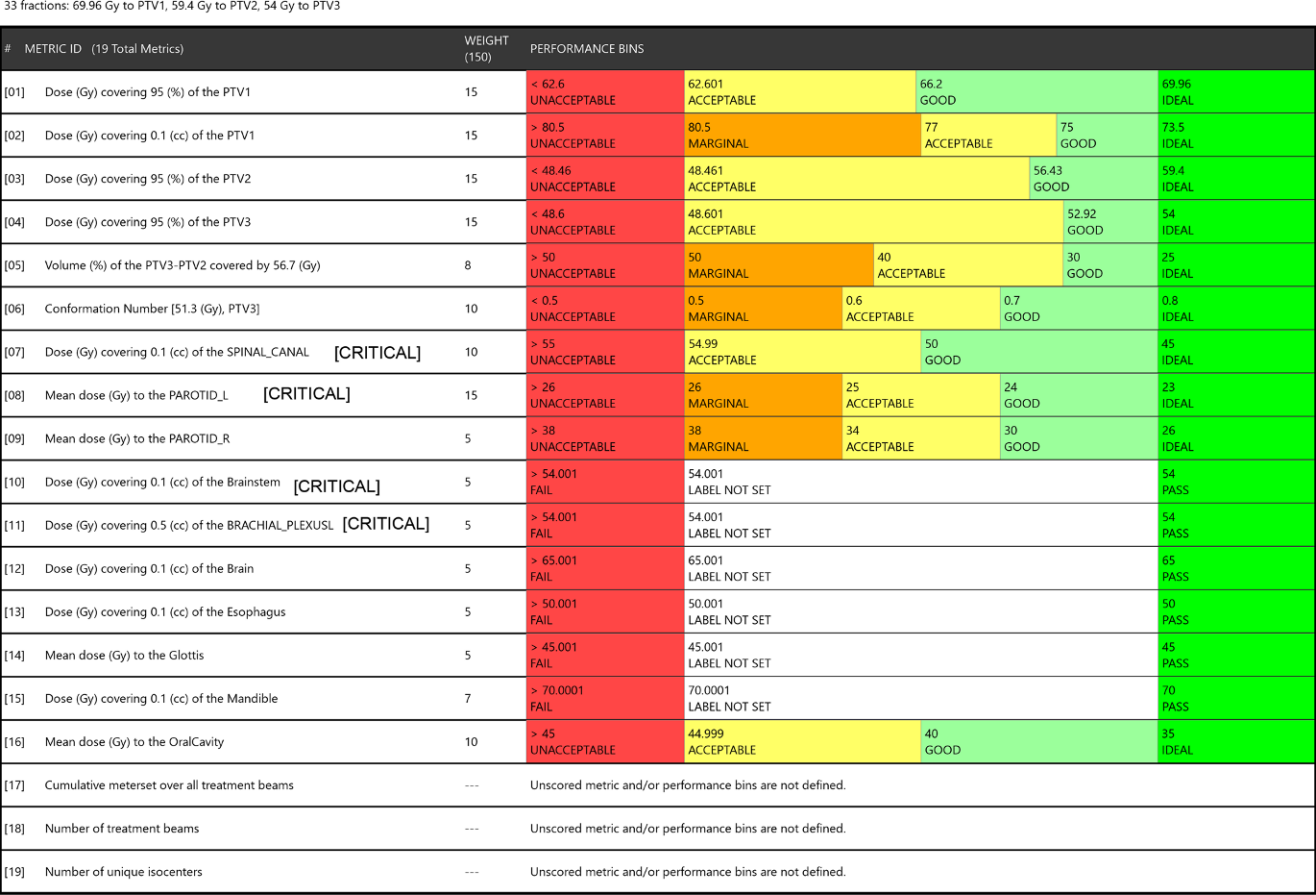
Scoring criteria as displayed on the ProKnow website created by ViewRay (Oakwood Village, OH, USA). *PTV1* high-risk volume, *PTV2* intermediate-risk volume, *PTV3* low-risk volume, *PTV* planning target volume

The scoring criteria for this challenge were based on conformity of the prescription doses, sparing of OARs, and coverage of PTVs. One of the evaluated parameters was the conformation number (CN), defined as TVRI * (TVRI) / (TV * VRI), with TVRI being the target volume (in this study PTV3) covered by the reference dose (cc), TV the total target volume (cc), and VRI the volume of the reference dose (cc). In total, 16 dose metrics were evaluated and are displayed in Fig. 2. For some metrics a range of points were available, depending on whether the dose achieved was deemed unacceptable, marginal, acceptable, good, or ideal in relation to what would be clinically acceptable. Other objectives for critical OARs were binary with pass or fail, scoring 0 points for any criteria not achieved (see Fig. 2 for scoring criteria). The sum of scores on all dose parameters results in a final score called the plan quality metric (PQM). Plans were defined as unacceptable if the total treatment time was longer than 20 min or if the PTV or OAR dose did not meet the constraints. 14 submissions were received and uploaded to this platform for analysis. Planners were also asked a few short questions about the amount of planning experience they had in general, and with the MRIdian system.

### Statistical analysis

All dose metrics have a threshold value that results in maximum points. For all dose parameters, the relative deviation from the maximum points threshold values were calculated. Differences with respect to the threshold were assessed with Wilcoxon signed-rank test. Besides dose metrics, beam-on time, treatment delivery time, number of beams, number of segments, number of monitor units (MU), and (ViewRay) planning experience were reported. Spearman’s ρ correlation coefficient was calculated to evaluate the correlation between each of the metrics. The total points for only PTV metrics and only OAR metrics were also included, to assess whether overall performance on PTV criteria corresponded to performance on OAR sparing. All statistics were performed in R (version 3.6.2, R Core Team 2020, Vienna, Austria), using the functions *wilcox.test* of the *stats* package and *rcorr* of the *Hmisc* package. A correlation coefficient above 0.7 was considered a strong correlation, above 0.5 was considered a moderate correlation, and below 0.5 was considered a weak correlation. *P*-values below 0.05 were considered significant.

## Results

**Fig. 3:**
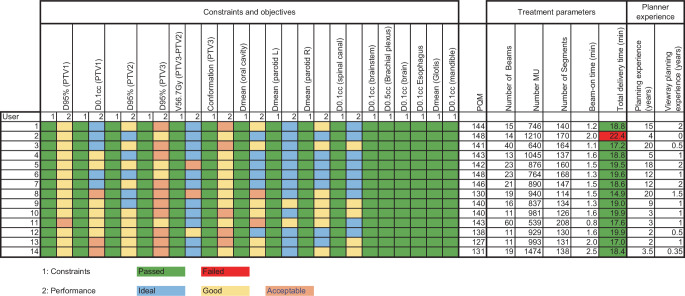
Descriptive statistics of all metrics evaluated in this study. *PTV1* high-risk volume, *PTV2* intermediate-risk volume, *PTV3* low-risk volume, *PTV* planning target volume

The detailed results for the 14 planners are included in Table 1 and Fig. 3. Each planner was able to achieve all the required constraints except for one planner for whom the total treatment delivery time exceeded the allowed time by 2.4 min (see Fig. 3). The median (range) number of beams used was 17.5 (11–60) and the median number of segments 139 (114–208), resulting in a total number of 909.5 MUs (539–1474), beam-on time of 1.5 min (0.8–2.5), and median total delivery time of 18.8 min (14.9–22.4). The median (range) IMRT planning experience for HNC of all users amounted to 7 years (2–20) and the ViewRay planning experience 1 year (0–2).

**Table 1:** Contains the descriptive statistics of all the metrics evaluated in this study

	Mean ± SD	Median [range]	Q0.25	Q0.75
PTV1 D_95%_ [Gy]	67.5 ± 1.2	67.0 [66.2–69.7]	66.7	68.5
PTV1 D_0.1cc_ [Gy]	74.1 ± 1.3	73.9 [72.0–76.6]	73.5	74.3
PTV2 D_95%_ [Gy]	59.0 ± 1.0	58.9 [57.5–60.8]	58.3	59.9
PTV3 D_95%_ [Gy]	52.4 ± 0.8	52.6 [50.7–53.5]	52.3	52.9
PTV3–PTV2 V_56.7_ _Gy_ [%]	22.3 ± 7.8	21.6 [8.3–35.8]	20.1	24.9
PTV3 conformation	0.7 ± 0.0	0.7 [0.7–0.8]	0.7	0.7
Spinal canal D_0.1cc_ [Gy]	44.0 ± 2.4	43.4 [39.0–48.8]	42.7	45.6
Parotid gland left D_mean_ [Gy]	22.0 ± 1.5	22.3 [17.8–23.8]	21.6	22.8
Parotid gland right D_mean_ [Gy]	26.4 ± 1.4	26.0 [24.4–29.1]	25.6	27.0
Brainstem D_0.1cc_ [Gy]	42.1 ± 4.0	42.1 [35.7–48.9]	39.2	43.8
Brachial Plexus D_0.5cc_ [Gy]	51.2 ± 1.6	51.6 [48.5–53.6]	49.9	52.2
Brain D_0.1cc_ [Gy]	48.5 ± 2.9	49.7 [43.2–52.4]	46.5	50.6
Esophagus D_0.1cc_ [Gy]	44.5 ± 2.7	44.4 [39.7–49.1]	42.8	46.4
Glottis D_mean_ [Gy]	42.3 ± 1.9	42.9 [39.1–44.9]	41.2	43.4
Mandible D_0.1cc_ [Gy]	59.7 ± 2.4	59.5 [56.9–64.8]	57.6	61.1
Oral cavity D_mean_ [Gy]	36.6 ± 3.5	36.0 [32.6–44.9]	34.1	39.2
Plan quality metric (max 150)	140.1 ± 6.5	141.5 [127.0–148.0]	138.5	143.8
OAR points (max 72)	70.3 ± 1.8	70.4 [67.5–72.0]	69.4	72.0
PTV points (max 78)	69.7 ± 5.5	71.4 [57.3–76.0]	66.7	73.4
Number of beams	21.1 ± 13.5	17.5 [11.0–60.0]	13.3	22.5
Number of MU	918.9 ± 232.7	909.5 [539.0–1474.0]	782.3	990.0
Number of segments	147.6 ± 24.2	139.0 [114.0–208.0]	131.8	163.0
Beam-on time [min]	1.5 ± 0.4	1.5 [0.8–2.5]	1.3	1.6
Total delivery time [min]	18.7 ± 1.7	18.8 [14.9–22.4]	17.8	19.6
Planning experience [years]	9.2 ± 6.9	7.0 [2.0–20.0]	3.1	14.3
ViewRay planning experience [years]	1.1 ± 0.6	1.0 [0.0–2.0]	0.6	1.4

*Q0.25* first quartile, *Q0.75* third quartile, *OAR* organ at risk, *PTV* planning target volume, *PTV1* high risk volume, *PTV2* intermediate risk volume, *PTV3* low risk volume, *MU* monitor units, *Dmean* mean dose, *min* time in minutes, *Dx* dose recieved by respective volume, *Vx* percentage volume receiving *x* Gy

**Fig. 4:**
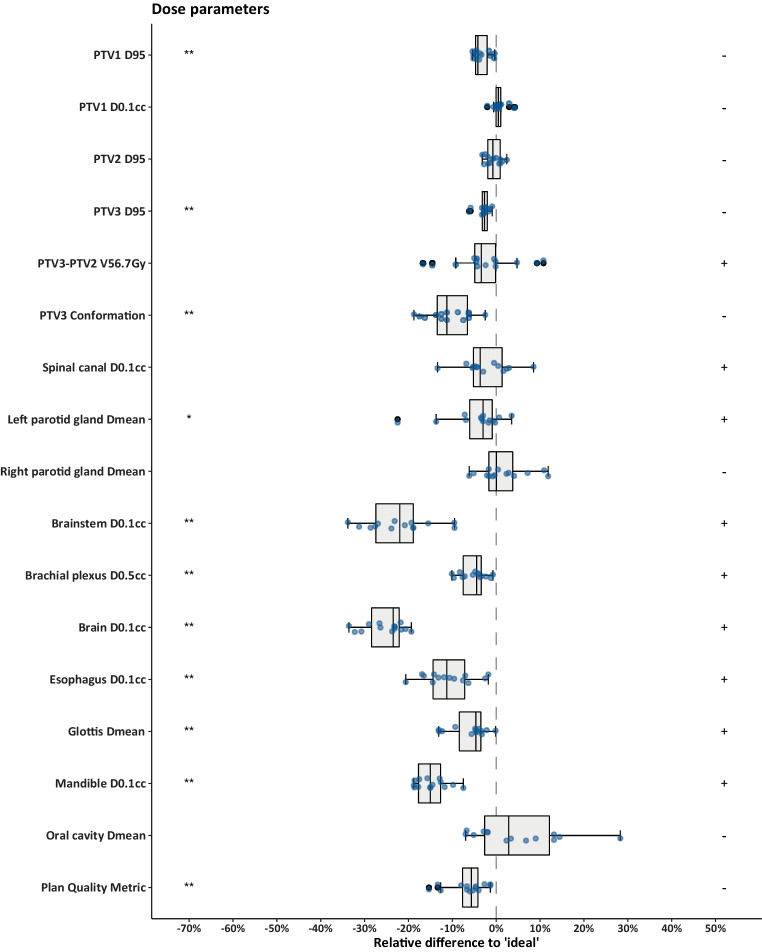
Relative difference of all evaluated metrics with respect to the value that results in maximum score. The +’and − signs on the right side indicate whether the median performance of all centers was better or worse than the ideal, respectively. *Asterisks* on the *left *indicate whether the scores were significantly different from the ideal score (*p < 0.05, **p < 0.01, ***p < 0.001). *PTV1* high-risk volume, *PTV2* intermediate-risk volume, *PTV3* low-risk volume, *PTV* planning target volume

The boxplot in Fig. 4 represents the relative deviation of the dose parameters with respect to the thresholds that result in maximum points for that metric. Note that the boxplot represents only ‘higher’ or ‘lower,’ which, depending on the metric, corresponds to either ‘better’ or ‘worse.’ The parameters for which the median performance of all centers was better than the threshold for maximum score are indicated with a plus sign in the boxplot, whereas a minus sign was used to indicate the opposite. The PTV1‑D_95%_, PTV1‑D_0.1cc_, PTV2‑D_95%_, and PTV3‑D_95%_ all deviate less than 7% from the optimal. For all OAR dose parameters, except the D_mean_ of the right parotid gland and the D_mean_ of the oral cavity, the median score of all centers was better than the threshold that results in maximum score. Six centers achieved the maximum number of points for the OAR dose parameters. None of the centers had an unacceptable performance on any of the metrics.

**Fig. 5:**
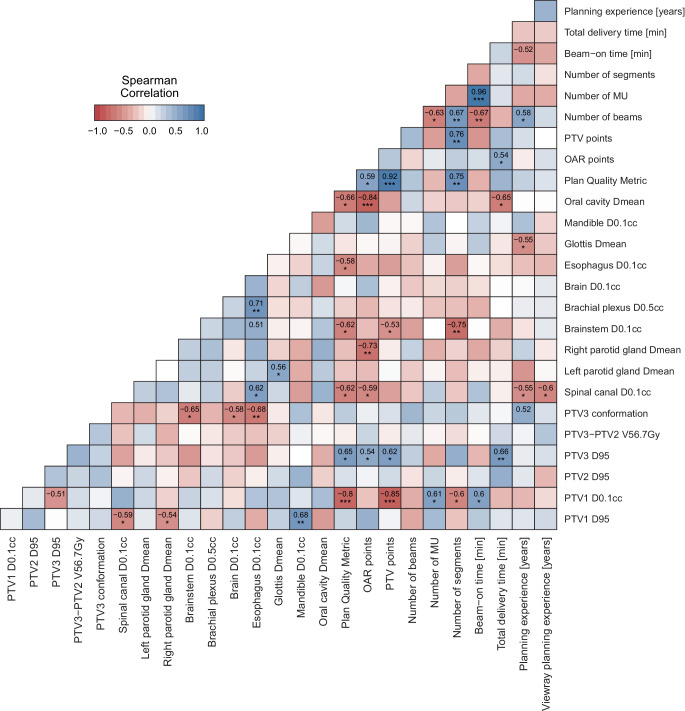
Correlation matrix representing Spearman’s ρ correlation coefficients for all evaluated metrics in this study. Only correlations that are considered moderate (*ρ* < −0.5 or *ρ* > 0.5) are displayed. *Asterisks* indicate significance (*p < 0.05, **p < 0.01, ***p < 0.001). *OAR* organ at risk, *PTV* planning target volume, *MU* monitor units, *PTV1* high-risk volume, *PTV2* intermediate-risk volume, *PTV3* low-risk volume

**Fig. 6:**
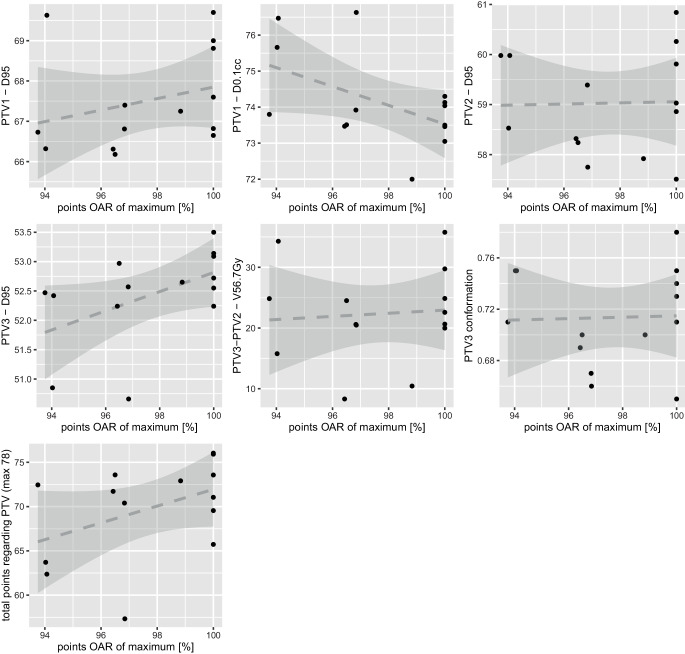
Plots indicating the relationship between different PTV dose parameters (y-axis) versus the total number of points achieved for OAR dose parameters (x-axis). The dotted line represents the linear fit and the *gray shaded areas* represent 95% confidence intervals. *OAR* organ at risk, *PTV* planning target volume, *PTV1* high-risk volume, *PTV2* intermediate-risk volume, *PTV3* low-risk volume

Fig. 5 shows the correlation matrix for all metrics evaluated in this study. Only correlation coefficients considered as moderate (i.e., lower than −0.5 or higher than 0.5) are displayed in the matrix. Significance levels are indicated as estimated by the *rcorr* function in R. The number of beams is moderately correlated to the number of MUs (*p* = 0.015), the number of segments (*p* = 0.0081), and the beam-on time (*p* = 0.0087). The number of MUs is also strongly correlated to the beam-on time (*p* < 0.0001). No correlation was observed between the number of beams and any of the dose metrics. The number of segments is strongly correlated to the brainstem‑D_0.1cc_ (*p* = 0.0022), and moderately correlated to the PTV1‑D_0.1cc_ (*p* = 0.025). The total delivery time is moderately correlated to two dose metrics: PTV3‑D_95%_ (*p* = 0.0095) and D_mean_ of the oral cavity (*p* = 0.011). The points total on the PTV metrics is only moderately correlated to one OAR metric: brainstem‑D_0.1cc_ (*p* *=* 0.049). The points total on OAR metrics is only moderately correlated to one PTV metric: PTV3‑D_95%_ (*p* *=* 0.047). The correlations between total OAR points and PTV metrics are shown in Fig. 6*.*

## Discussion

In recent years there have been a few comparative multicenter planning studies, both for stereotactic body radiotherapy (SBRT) [15–18] and for IMRT, even for HNC [19]. These studies helped to share experiences for specific treatment indications, and in some cases even led to long-term plan improvement through targeted intervention, i.e., sharing the best plans regarding sparing OARs with the other centers [19]. However, to the best of our knowledge, this is the first study comprising ViewRay TPS for HNC. Planning HNC treatment on such platforms as the MRIdian system presents some technical difficulties compared to the current standard. In most high-volume centers, HNC treatments are planned with volumetric modulated arc therapy (VMAT), which allows the delivery of a highly conformal dose distribution in a very short timeframe [20, 21]. The sole technique available with the MRIdian system is step-and-shoot IMRT. The delivery time is therefore much longer than on a standard linac (15–20 min), mainly due to the slow MLC motion and the gantry rotation speed. Additional limitations originate from limited beam angles (dead zone 30–33 degrees and recommended avoidance of couch edges), the lack of a collimator preventing the use of the MLC in different directions, and no option to use more efficient delivery techniques such as sliding window.

In the present planning study, we have demonstrated that treatment plans of high quality and complexity for treating HNC on the MRIdian platform resulting in reasonable treatment delivery time can be achieved with sufficient IMRT planning experience and through increasing the number of segments used for a particular plan.

Recently developed hybrid machines consisting of a linear accelerator and integrated MRI (MR linacs) could allow a) better visualization of tumor and organs at risk, such as parotid glands during patient positioning and daily treatment; b) daily imaging without additional radiation exposure; c) reduction of established safety margins for the treatment volumes; and, finally, d) frequent adaptation of target volumes according to changes in patient weight and tumor anatomy during the radiotherapy course. These procedures would facilitate a high-precision treatment and help reduce dose exposure of critical structures. MR linacs are already in use in several centers worldwide and published studies have shown that the quality of MR linac plans achieved was sufficient and not inferior to other linacs for various anatomical sites and diseases [21–23]. Authors from different institutions showed that MR-guided radiotherapy is tolerated by the patients [24], compared MR-based planning with conventional linac-based stereotactic radiotherapy [25], and discussed potential advantages [26]. These studies mainly reported the clinical feasibility of stereotactic body radiation therapy MR-guided radiotherapy, but data for complex and large target volumes such as used for curative, conventional fractionated therapy like head and neck treatments are still very rare, and all of the published studies for HNC have been conducted either in non-linac (cobalt) platforms, or had no specific focus on plan optimization [27, 28]. These first applications appear encouraging: Chen et al. have shown feasibility of MR-guided radiotherapy for both primary cases and in the challenging situation of re-irradiation of recurrent HNC [27, 29]. MR-adapted planning could allow for repetitive and more precise plan adaptations, taking into account such challenges as weight loss, inter-fractional differences, and organ and tumor motion [30–32], which are all important problems of head and neck radiotherapy. Advantages in this regard might allow for safer and personalized radiotherapy in the future. The MRIdian® technology, which was used in this study, combines 0.35 T MRI with a multileaf-collimator linac and has already been described in detail before [9, 33–35]. Furthermore, various methods and procedures for quality assurance of the system are meanwhile also well established [31, 36–38].

The results of this study could demonstrate that IMRT plans of relatively high quality can already be developed after a median ViewRay user experience of about 1 year if the planner has gained sufficient experience with IMRT for HNC in the past (in the present study a median of 7 years). All of the plans developed would have been applicable and acceptable in clinical routine without any compromises in PTV or OARs constraints. Interestingly, 6/14 centers could even achieve the maximal quality score possible for OAR sparing. None of the centers were able to achieve an ideal conformation number of 0.8, but 10/14 centers achieved good performance within the range (0.7–0.8), whereas 4/14 centers achieved acceptable performance within range (0.6–0.7). Although none of the plans had a marginal or unacceptable conformation number, the moderate, negative correlation between conformation number and several OARs shows the challenge of target coverage and simultaneous OAR sparing. Other interesting correlations between dose parameters and plan characteristics were observed in this study (Fig. 5). Planning experience was moderately correlated with improved planning results for some specific OARs like spinal cord D_0.1cc_ (ρ = −0.55) or glottis D_mean_ (ρ = −0.55). In line with this observation, previous studies have demonstrated improved outcomes with increasing planning experience for patients treated with IMRT for HNCs [39, 40], and other malignancies [41]. Intriguingly, the planning experience did not correlate with the overall points scored in the present challenge. A possible explanation for this phenomenon could be that all of the participants of this study originated from high-volume centers, where the number of patients treated per time unit and the resulting experience are already high [40]. Another interesting observation of the present study was the significant correlation of the number of segments used and both the “PTV points” (ρ = 0.76) and the PQM (ρ = 0.75), making this the most important factor for plan quality. Although a higher number of segments could generally correlate with longer delivery times as has been shown before [42], this was not the case with the present example. The relatively high number of beams used by all of the participants (median 17.5) resulting in long delivery times could explain the lack of significant association of segments and delivery duration. However, a slight reduction of the segments and increase of beam angles might allow for faster treatment in future planning [42]. Interestingly, increasing the number of beams was associated with an increased segment number (ρ = 0.67), even though the number of total segments can be limited in the planning system. Sutton et al. recommends not more than 3–6 segments per beam for good plan quality of IMRT plans [43], which is clearly less than the numbers used here (median 139 segments for 17.5 beams). The treatment delivery time remains an issue of step-and-shoot IMRT and of the present platform: with a median of 18.8 min, it is longer compared to other modern IMRT and especially VMAT platforms [20]. This was the only parameter not met by 100% of the participants. As the median beam-on time of 1.5 min for a median of 909 MUs was comparable or even better than Halcyon and True-Beam plans [44], the slower application is mainly attributed to the MLC parameters such as leaf speed. An increase in the number of beams was actually associated with a reduction in the total monitor units (ρ = −0.63), and thus with a reduced total delivery time. An upgrade with faster MLC movement would surely facilitate wider implementation of the platform in clinical routine. Patients with HNC in advanced stages, with rapidly reacting tumors like human papilloma virus-positive oropharyngeal or Epstein-Barr Virus-positive nasopharyngeal carcinoma, with irradiation of both neck sides, with the PTV in close proximity to various OARs, and with moving targets such as small laryngeal carcinomas could possibly benefit the most from treatment on MR linac platforms due to the possibility of online imaging during treatment and repeated adaptations. However, intrafractional and interfractional variations for this cancer type do not occur as frequently and unexpectedly as in moving abdominal organs or the pelvic region. Furthermore, only conventional fractionation or generally low-dose-per-fraction regimens are mostly used for this indication. The potential added value of online adaptation should be considered and carefully weighed against a prolonged treatment time. An offline and less frequent than daily adaptation could be sufficient for most HNC cases. Taken together, the planning time is not as crucial for this indication as, in contrast to SBRT applications, planning will be mostly done offline. Much more important for applying a standard regimen of radiotherapy for HNC over 7 weeks and over 30 fractions will be the treatment and beam delivery time. Future improvements in this direction, like integrating sliding window and VMAT techniques in the MR linac, will surely improve patient comfort and compliance. Due to this reason, we decided to assess these last parameters in this study.

There are a few limitations of this study. First or all, the present analysis does not focus on a clinically challenging situation, e.g., SBRT for spinal metastases [15], nor on implementation of society guidelines [16], nor on optimizing OAR sparing [19]. The focus of the present work was to investigate the application and the features of the modern technology of MR linac and a relatively novel planning system for the new indication of head and neck cancer and to generate hypotheses for future plan optimization. The number of centers that participated is probably enough for such purposes, as in the Dutch planning study for HNC a similar number of centers (15) was enough to be even practice changing [19].

Another possible shortcoming of this multicenter study could be the omission of calculating a complexity index (CI) for all plans as described by several authors before for planning studies of IMRT or SBRT [45]. However, Hernandez et al. stressed the importance of CI implementation, especially for comparison of multicenter results when using different planning systems, as the differences when comparing other quality metrics for plans developed in the same planning system were not so pronounced [46]. Since all the participants in this study used the same planning system, the complexity index was not evaluated. Also, as only one case was included, the results might not be applicable to all HNC plans.

Furthermore, prior to actual treatment delivery, quality assurance (QA) is required in order to avoid dose delivery errors [47]. Nevertheless, the evaluation of in vivo dosimetry was not within the scope of this study, so no measurements of the actual plans were performed.

Despite these limitations, this is the first study providing treatment planning recommendations for treating HNC on the MRIdian platform as a result of a multicentric effort of experienced users. In addition, this is one of the first studies demonstrating feasibility and quality of planning for HNC on the MRidian platform and providing recommendations for the numbers of beams and segments to start with.

## Conclusion

Clinically acceptable and excellent treatment plans for HNC were achieved by most users on the MRIdian platform. The quality of these plans can be optimized through implementation of a higher number of segments and increasing experience of the planner and can thereby achieve clinically acceptable results. Inauguration of MR linacs for routine clinical treatment of HNC patients appears already feasible.

## Supplementary Information

Supplementary Fig. 1: Patient positioning for head and neck treatment on the MRIdian including coils and thermoplastic mask placement.

Supplementary Table 1: Hard PTV and OAR constraints for treatment planning given to participants. If constraints were not fulfilled zero points would be awarded for that particular structure.
