# Supplementary figures and images for: Head and neck radiotherapy on the MR linac: a multicenter planning challenge amongst MRIdian platform users
Source: Strahlenther Onkol. 2021 Apr 23;197(12):1093–103. doi: 10.1007/s00066-021-01771-8 (PMC8604891; doi:10.1007/s00066-021-01771-8)

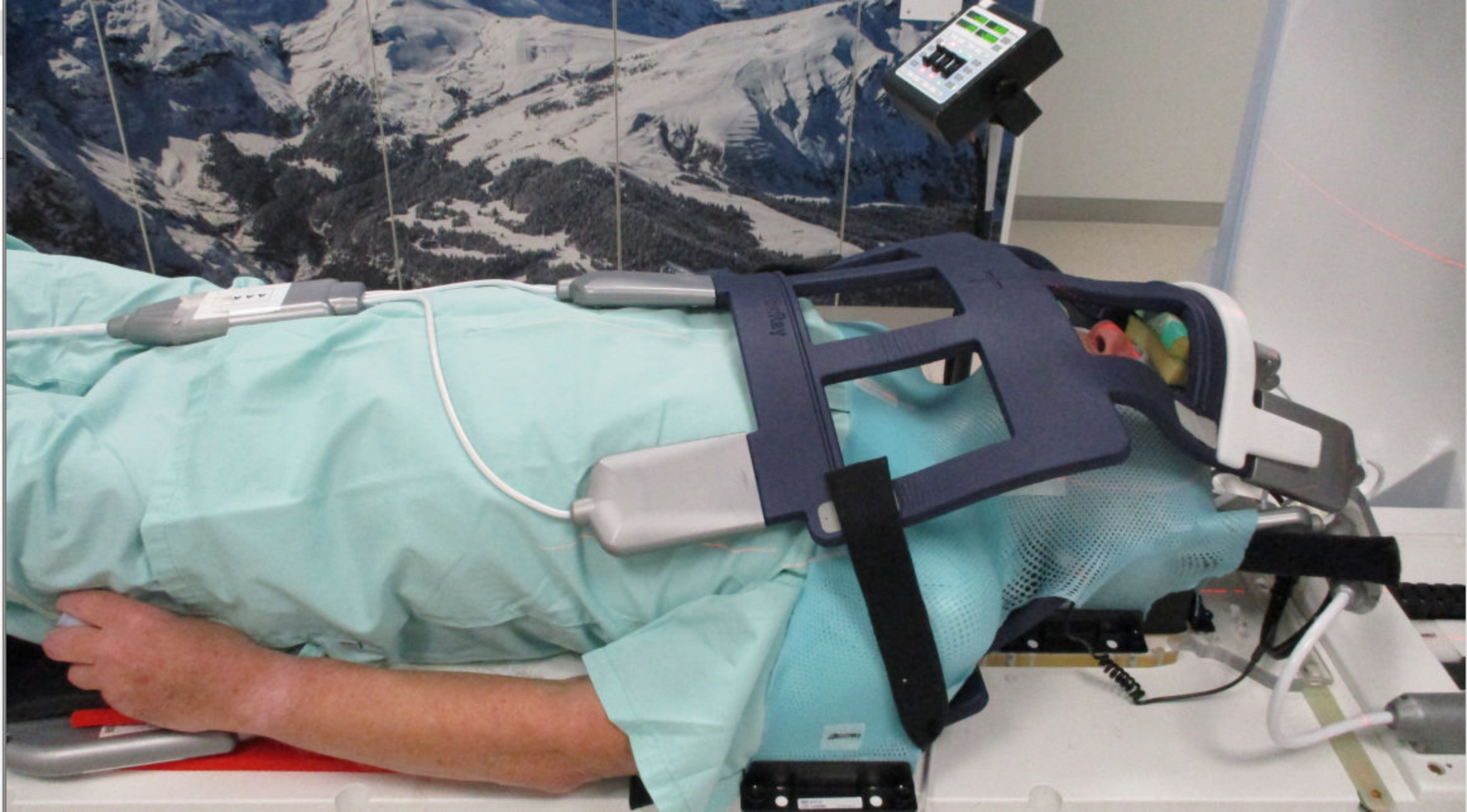

Supplement: Supplementary file 1 — Supplementary Fig. 1: Patient positioning for head and neck treatment on the MRIdian including coils and thermoplastic mask placement. [file 66_2021_1771_MOESM1_ESM.pdf]
