# Supplementary material for: Head and neck radiotherapy on the MR linac: a multicenter planning challenge amongst MRIdian platform users
Source: Strahlenther Onkol. 2021 Apr 23;197(12):1093–103. doi: 10.1007/s00066-021-01771-8 (PMC8604891; doi:10.1007/s00066-021-01771-8)
Supplement: Supplementary file 2 — Supplementary Table 1: Hard PTV and OAR constraints for treatment planning given to participants. If constraints were not fulfilled zero points would be awarded for that particular structure. [file 66_2021_1771_MOESM2_ESM.docx]

***Supplementary Table 1*** *Hard constraints given to participants. If not fulfilled zero points would be awarded for that particular structure.*

| **Structure** | **Constraint** |
| --- | --- |
| **PTV1 (69.96Gy)** | D95% > 62.6Gy |
| **PTV1 (69.96Gy)** | D0.1cc < 80.5Gy |
| **PTV2 (59.4Gy)** | D95% > 48.46Gy |
| **PTV3 (54Gy)** | D95% > 48.6Gy |
| **PTV3-PTV2** | V56.7Gy <50% |
| **Conformation Number PTV3 (51.3Gy)** | >0.5 |
| **Spinal Canal** | D0.1cc < 55Gy |
| **Parotid_L** | Mean Dose < 26Gy |
| **Brainstem** | D0.1cc < 54Gy |
| **Brachial_PlexusL** | D0.5cc < 54Gy |
| **Brain** | D0.1cc < 65Gy |
| **Esophagus** | D0.1cc < 50Gy |
| **Glottis** | Mean Dose < 45Gy |
| **Mandible** | D0.1cc < 70Gy |
| **Oral Cavity** | Mean Dose < 45Gy |
